# Ultrastructural Abnormalities in CA1 Hippocampus Caused by Deletion of the Actin Regulator WAVE-1

**DOI:** 10.1371/journal.pone.0075248

**Published:** 2013-09-25

**Authors:** Diána Hazai, Róbert Szudoczki, Jindong Ding, Scott H. Soderling, Richard J. Weinberg, Péter Sótonyi, Bence Rácz

**Affiliations:** 1 Department of Anatomy and Histology, Faculty of Veterinary Science, Szent István University, Budapest, Hungary; 2 Department of Ophthalmology, Duke Eye Center, Duke University, Durham, North Carolina, United States of America; 3 Departments of Cell Biology and Neurobiology, Duke University, Durham, North Carolina, United States of America; 4 Department of Cell Biology & Physiology and Neuroscience Center, University of North Carolina, Chapel Hill, North Carolina, United States of America; Institute for Interdisciplinary Neuroscience, France

## Abstract

By conveying signals from the small GTPase family of proteins to the Arp2/3 complex, proteins of the WAVE family facilitate actin remodeling. The WAVE-1 isoform is expressed at high levels in brain, where it plays a role in normal synaptic processing, and is implicated in hippocampus-dependent memory retention. We used electron microscopy to determine whether synaptic structure is modified in the hippocampus of WAVE-1 knockout mice, focusing on the neuropil of CA1 stratum radiatum. Mice lacking WAVE-1 exhibited alterations in the morphology of both axon terminals and dendritic spines; the relationship between the synaptic partners was also modified. The abnormal synaptic morphology we observed suggests that signaling through WAVE-1 plays a critical role in establishing normal synaptic architecture in the rodent hippocampus.

## Introduction

Most excitatory neurons in the mammalian forebrain have a long axon, and several shorter dendrites covered with spines. These dendritic spines are the primary target of glutamatergic axon terminals; modifications in spine shape and size are associated with multiple forms of long-term synaptic plasticity [1]. Spines are rich in actin, their principal cytoskeletal element [2,3,4,5]. Actin is also found in presynaptic axon terminals, where it can modulate the organization of the different pools of synaptic vesicles [6,7]. For example, by creating a barrier between the reserve pool and the presynaptic active zone, F-actin may lower release probability [8]; conversely, through interaction with synapsins, actin can facilitate transfer of vesicles from the reserve pool into the readily-releasable pool [9]. Thus, the actin cytoskeleton is important for both pre- and postsynaptic function.

Extensive research in model systems has shown that the actin cytoskeleton is dynamically controlled via an elaborate network of biochemical cascades [10,11]. A key upstream component of this cascade is the Rho/Rac family of small GTPases [12,13,14,15,16,17,18], which are also involved in neuronal proliferation and migration during development, and help to regulate synaptic plasticity in the mature brain [19]. Signaling through these GTPases is relayed to the actin cytoskeleton by the WAVE (Wiskott-Aldrich syndrome verprolin homology) family of scaffolding proteins [20,21,22,23]. WAVE contains multiple protein-interaction domains: the N-terminal SCAR-homology domain regulates Rac signaling [24], and a central proline-rich region can interact with SH3 domain-containing proteins, whereas the C-terminal Verprolin-Cofilin-Acidic domain plays a key role in activation of the Actin-Related Protein 2/3 (Arp2/3) complex, which mediates nucleation and branching of F-actin filaments [20]. Thus, WAVE provides a platform to assemble multiple molecules that can interact to modulate remodeling of the actin cytoskeleton.

While our knowledge of WAVE signaling derives mainly from studies in model systems, accumulating evidence points to an important role for WAVE-mediated signaling also in neurons [21,25,26,27]. *In vitro* evidence suggests that WAVE-1 (the major isoform in brain [28]) is required for the formation, maintenance, and activity-dependent reorganization of dendritic spines; moreover, loss of WAVE-1 function reduces spine number and alters spine shape in cultured hippocampal neurons [29]. WAVE-1 is also found in axon terminals, where *in vitro* experiments suggest an important role in neurite growth and formation of axonal filopodia [21,29,30,31,32,33,34]. Behavioral and electrophysiological studies in mutant mice show that WAVE-1 deletion leads to disrupted synaptic plasticity in the hippocampus, and impairs hippocampus-dependent learning and memory [21,27]. However, it remains unclear whether genetic deletion of WAVE-1 affects the architecture of synapses in the intact hippocampus. We here use quantitative electron microscopy to investigate alterations in the CA1 neuropil caused by genetic ablation, finding that loss of WAVE-1 protein disrupts the architecture of both axon terminals and dendritic spines.

## Materials and Methods

The WAVE-1 knockout (KO) mice used in this study have been described previously [21,27]. Experimental animals were littermates from heterozygous pairings. All mice were housed in Duke University’s Division of Laboratory Animal Resources facilities. All procedures were conducted with protocols approved by the Szent István University (permit numbers: 22.1/2060/3/2011, MÁB 18/2011) and Duke University Institutional Animal Care and Use Committees (permit number: A288-11-11) in accordance with Hungarian Animal Health and Welfare Committee and U.S. National Institutes of Health guidelines. All efforts were made to minimize the number of animals, and to minimize animal stress, suffering, and discomfort.

### Preparation of tissue

Experiments were carried out on 60-65 day old C57BL/6 WAVE-1 *KO* mice and wild-type (wt) littermate controls of both sexes. Animals were deeply anesthetized with pentobarbital (60 mg/kg i.p.), then perfused intracardially with saline, followed by a mixture of depolymerized paraformaldehyde (4%) and glutaraldehyde (0.2%) in 0.1 M phosphate buffer pH 7.4 (PB). Sections were postfixed in 0.5-1% osmium tetroxide in 0.1 M PB for 35-45 min and stained *en bloc* with 1% uranyl acetate for 1 h. After dehydration in ascending ethanol series and propylene oxide, sections were infiltrated with Epon/Spurr resin (EMS) and flat-mounted between sheets of Aclar within glass slides. For single section analysis seventy nm sections were cut and mounted on 300 mesh copper grids; for three dimensional reconstructions, fifty nm serial sections were mounted on Formvar-coated single slot grids (EMS) and contrasted with uranyl acetate and Sato’s lead. We used the freely available Reconstruct software (see http:// http://synapses.clm.utexas.edu/tools/reconstruct/reconstruct.stm) to reconstruct spines from serial sections [35].

### Data analysis

Material was examined in a JEOL T1011 electron microscope at 80 KV; randomly-selected images from stratum radiatum of CA1 were collected with a MegaView (Soft Imaging System) 12 bit 1024x1024 CCD camera at a uniform magnification, resulting in 32.98 µm^2^ fields. Data collection and measurements were performed in a blinded manner. Areas, perimeters and profile circularity (4π*area/perimeter^2^) were measured using the engine provided by NIH ImageJ [36]. We used the Wilcoxon rank-sum text to assess the possible statistical significance of differences between *wt* and *KO* datasets, since this robust non-parametric test does not assume normality in the underlying population.

## Results

We studied the stratum radiatum of CA1 hippocampus in both *wt* (n=3, M5235, M5236 and M5264) and WAVE-1 *KO* mice (n=3, M5229, M5233 and M5266), examining axon terminals, dendritic spines, and synaptic contacts. Results were generally consistent among animals (Table S1). We detected no obvious differences between measurements from males and females [37], and therefore pooled data from both sexes in our analysis.

### Presynaptic axon terminals

Analysis of single sections collected from CA1 stratum radiatum revealed that the genetic lesion led to a slight (~8%) increase in density of axon terminals, 86.2 ± 1.6 (SEM) per 100 µm^2^ in the *KO* (n=1990 terminals from 70 fields from 3 animals), versus 79.5 ± 1.6 per 100 µm^2^ in the *wt* (n = 2334 terminals from 89 fields, 3 animals, P < 0.05, Wilcoxon; median density of 86.4 vs 78.8 terminals per 100 µm^2^). We asked how many of these terminals establish synaptic contacts with spines (counting only terminals that made synaptic contact with dendritic spines, as defined by the presence of a clearly visible synaptic cleft and a postsynaptic density in the partner spine), finding that their proportion was reduced by ~ 33%: in CA1 stratum radiatum neuropil from *KO* mice only 50.4% of the terminals made an axospinous synaptic contact in the plane of section (n=1003 terminals out of 1990), while in *wt* mice, 75.2% of the terminals contacted spines (n=1756 terminals out of 2334). To assess significance, we asked what fraction of terminals contacted spines in each of 70 fields from *KO* animals and 89 fields from *wt* animals, finding P < 0.001 (median for *KO* = 50.0%; for wt = 75.7%). We conclude that while deletion of WAVE-1 slightly increased the number of terminals in CA1 stratum radiatum, it substantially reduced the fraction of terminals that make visible synapses with spines.

**Figure 1:**
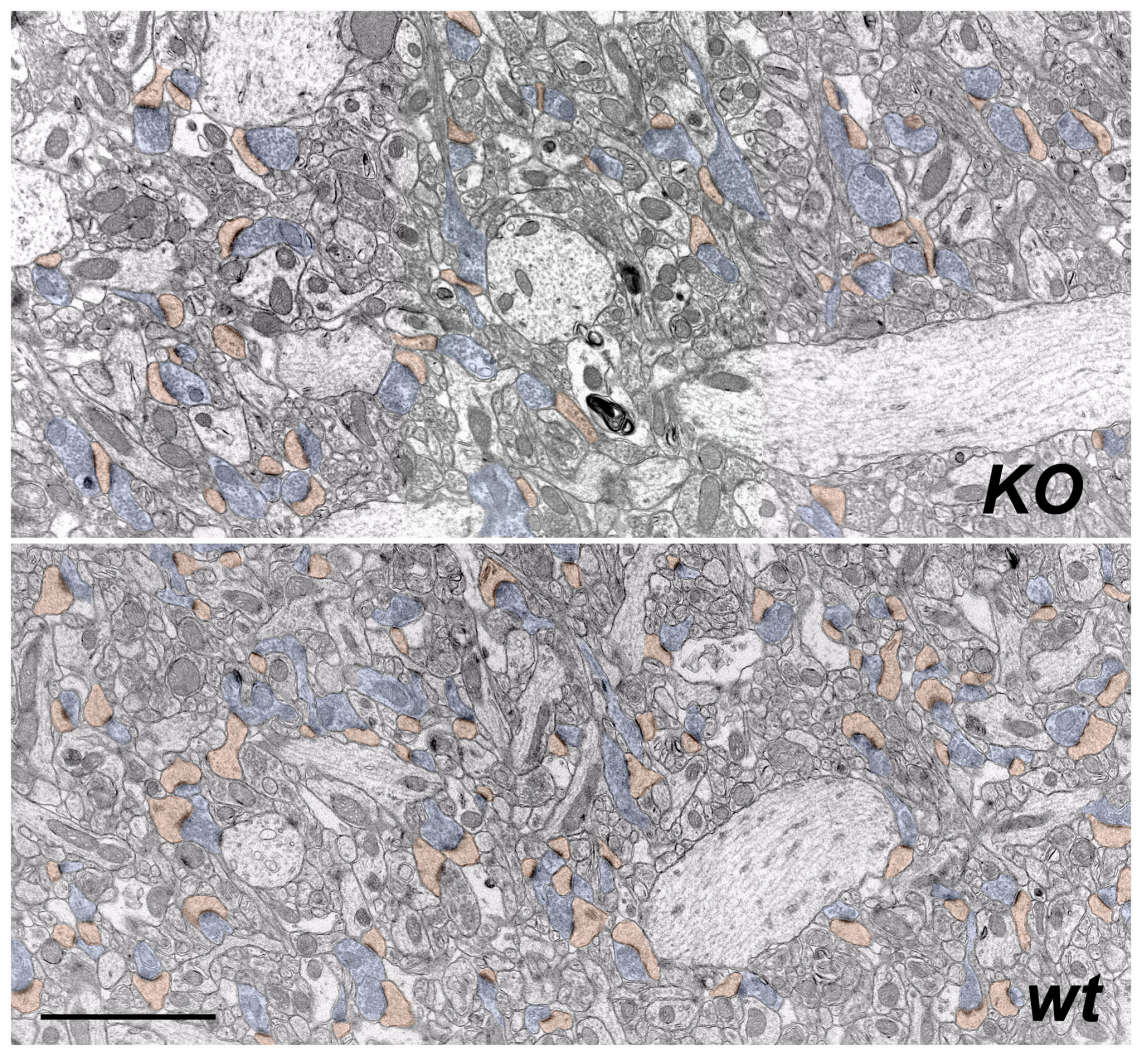
Overview of ultrastructural changes associated with deletion of WAVE-1. Representative low-magnification electron micrographs of synaptic neuropil in stratum radiatum of CA1 hippocampus, showing postsynaptic spines (orange) and presynaptic boutons (blue) in material from *KO* (A) and *wt* mice (B). Scale bar: 5 µm.

**Figure 2:**
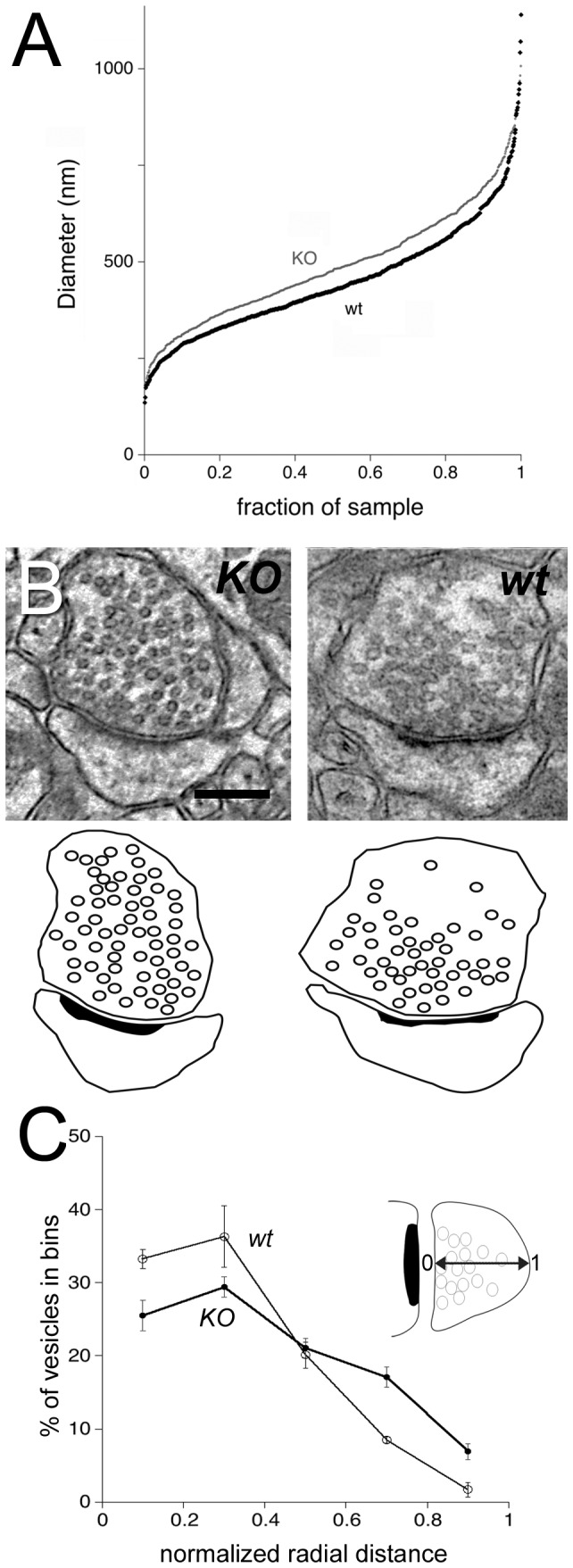
WAVE-1 affects size of presynaptic terminals and organization of synaptic vesicles. **A**. Cumulative plot shows the distribution of the mean diameter (defined as sqrt(area*4/π)) of terminals in KO (grey circles) and *wt* (black diamonds) CA1 KO terminals are generally larger than *wt* terminals. **B**. Electron micrographs (upper panel) and corresponding line drawings (lower panel) illustrate organization of synaptic vesicles within an axon terminal from a *KO* mouse (left), compared to *wt* control (right). Micrographs are from stratum radiatum of CA1 hippocampus. Synaptic vesicles are more numerous and lie farther from the active zone in *KO* mice, compared to *wt*. Scale bar: 200 nm. **C**. Quantitative analysis of the organization of vesicles in *KO* mice, versus *wt* controls. To combine data from terminals of different sizes, the distribution of vesicles was normalized (see inset): 0 corresponds to a vesicle lying directly at the presynaptic membrane, and 1 to a vesicle lying at the opposite non-synaptic membrane along an axis perpendicular to the synapse. Black circles (representing positions of *KO* vesicles in terminals) tend to lie farther from the active zone than white circles (representing positions of the *wt* vesicles). Vertical bars are standard errors (N = 3 animals for each genotype).

Qualitative examination suggested that the morphology of terminals is subtly altered in the *KO* mice (Figure 1). Quantitative analysis confirmed this impression. We found that terminals from mutant mice were ~18% bigger (Figure 2A; terminal area of 0.206 ± 0.004 µm^2^ in the *KO* (n = 750 terminals), versus 0.174 ± 0.004 µm^2^ in the *wt* (n = 772); medians of 0.177 vs 0.142; P < 0.001). Furthermore, these bigger terminals contained ~24% more synaptic vesicles than terminals from the *wt* (26.8 ± 1.5 synaptic vesicles/terminal in the *KO* (n= 77 terminals from 3 animals); 21.6 ± 1.1 synaptic vesicles/terminal in the *wt* (n = 77 terminals from 3 animals); P < 0.02).

Presynaptic vesicles in the *KO* seemed to be less tightly associated with the active zone than in the *wt*, whose vesicles typically concentrated at the active zone, becoming sparse at the periphery of the axon terminal (Figure 2B). To analyze their relative position, we measured the distance of vesicles from the synaptic membrane of the active zone, confirming that vesicles lay considerably further from the active zone in *KO* animals (180 ± 6 nm; n = 596 vesicles) than in the *wt* (136 ± 4 nm; n = 574); medians of 147 vs 111; P < 0.001. However, *KO* terminals were larger than those from *wt* mice, potentially confusing the issue. We controlled for this difference by computing a normalized distribution of synaptic vesicles, such that a vesicle directly touching the presynaptic membrane at the active zone would have a normalized radial distance of 0, and a vesicle at the opposite side of the plasma membrane would have a normalized distance of 1.0 (see inset in Figure 2C). This analysis confirmed our subjective impression, showing that vesicles in the *KO* lay at a mean distance of 0.40 normalized units, while synaptic vesicles in the *wt* lay closer to the synapse, at a mean normalized distance of 0.29 (Figure 2C; medians of 0.37 vs 0.29; *P* < 0.001). Thus, bigger axon terminals in the WAVE-1 *KO* animals contained significantly more vesicles, which distributed abnormally within the terminal.

### Postsynaptic dendritic spines

**Figure 3:**
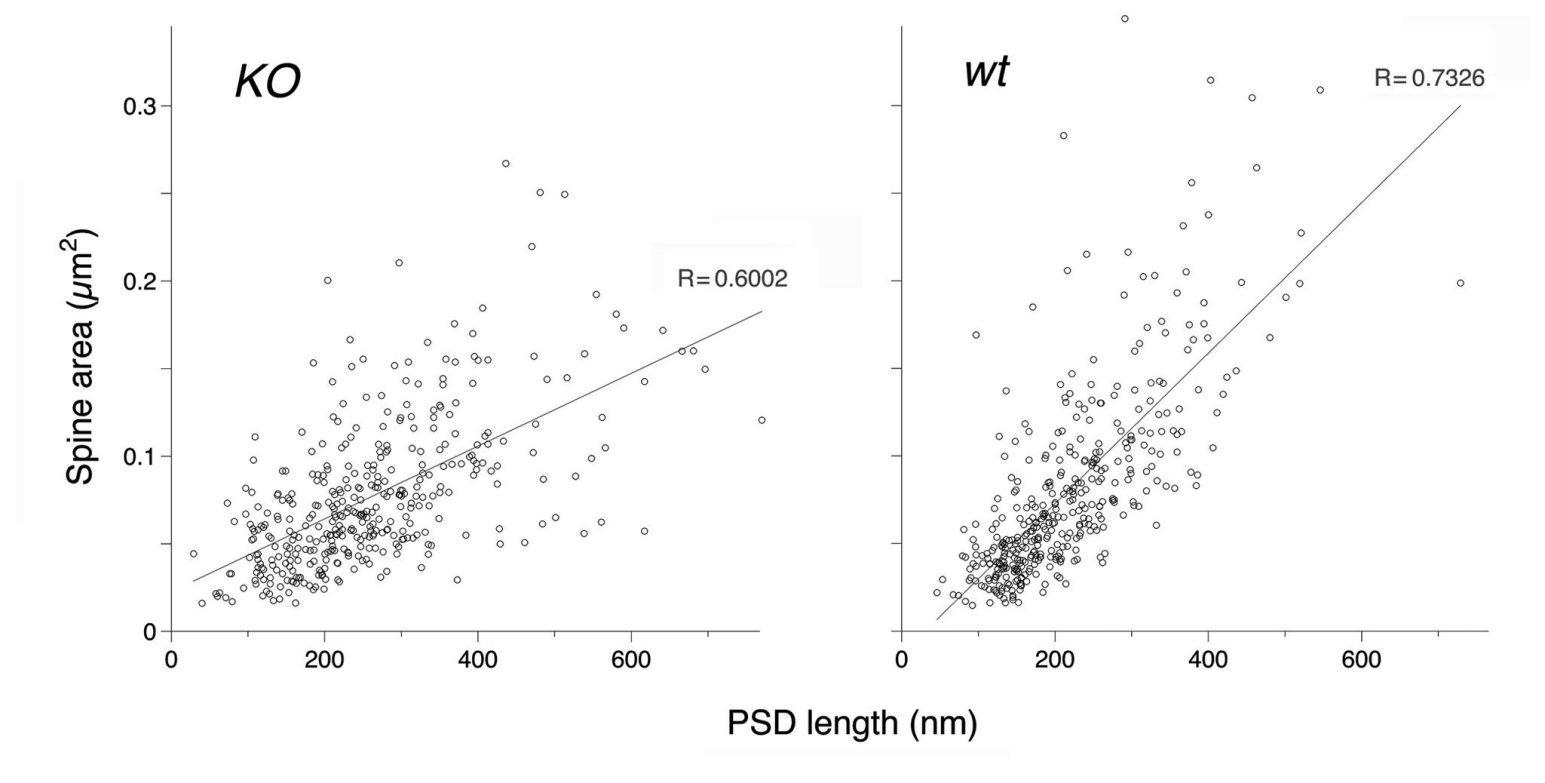
WAVE-1 affects relationship between PSD size and spine size. Scatterplots show the area of spine profiles as a function of PSD length, in CA1 stratum radiatum of *KO* (left) and *wt* hippocampus (right). Linear regression analysis demonstrates a weaker correlation between spine area and PSD length in the *KO* (R = 0.60) than the *wt* material (R = 0.73).

Consistent with previously-published evidence from light microscopy of *in vitro* material that suggested a reduction in spine number as a result of WAVE-1 loss, we found a marked (~30%) reduction in the number of postsynaptic spines in CA1 stratum radiatum from *KO* mice (*KO*, 42.8 ± 1.2 spines/100 µm^2^, n = 70 fields; *wt*, 60.7 ± 1.5 spines/100 µm^2^, n = 80 fields; P < 0.001; Figure 1). We compared the size of spines from *KO* animals with those from *wt* (n = 384 spines in *KO* and 395 in *wt*), finding no significant differences in area (*KO*, 0.077 ± 0.002 µm^2^; *wt*, 0.079 ± 0.002 µm^2^; P > 0.4). In contrast, the spine *perimeter* was significantly increased in *KO* mice (mean perimeter for *KO*, 1221 ± 22 nm; *wt*, 1133 ± 21 nm; P < 0.002). Likewise, the length of postsynaptic densities (PSDs) as seen in single sections was significantly longer in *KO* spines (260 ± 6 nm) compared to *wt* spines (216 ± 5 nm; P < 0.001). Previous work using serial-section microscopy shows that larger spines tend to have larger PSDs [38]. Accordingly, we analyzed the relationship between spine head area and PSD length in our material. Randomly-selected single sections of spines from CA1 stratum radiatum of the *KO* mice exhibited a positive correlation between spine size and PSD length (r^2^ = 0.36), but there was a considerably stronger correlation for spines from *wt* mice (r^2^ = 0.54; Figure 3).

That spine perimeter increased while spine area was unchanged in the *KO* mice suggests spine profiles from *KO* mice were less round. To test this, we computed the "circularity" of randomly selected spine head profiles from single sections (a value of 1.0 indicates a perfect circle, and 0 indicates a completely flattened shape; see methods for details). We found that spines in the *KO* mice were significantly less circular (0.65 ± 0.01) than spines from the *wt* stratum radiatum (0.74 ± 0.01; P < 0.001), implying that *KO* animals have flattened or elongated spine heads.

**Figure 4:**
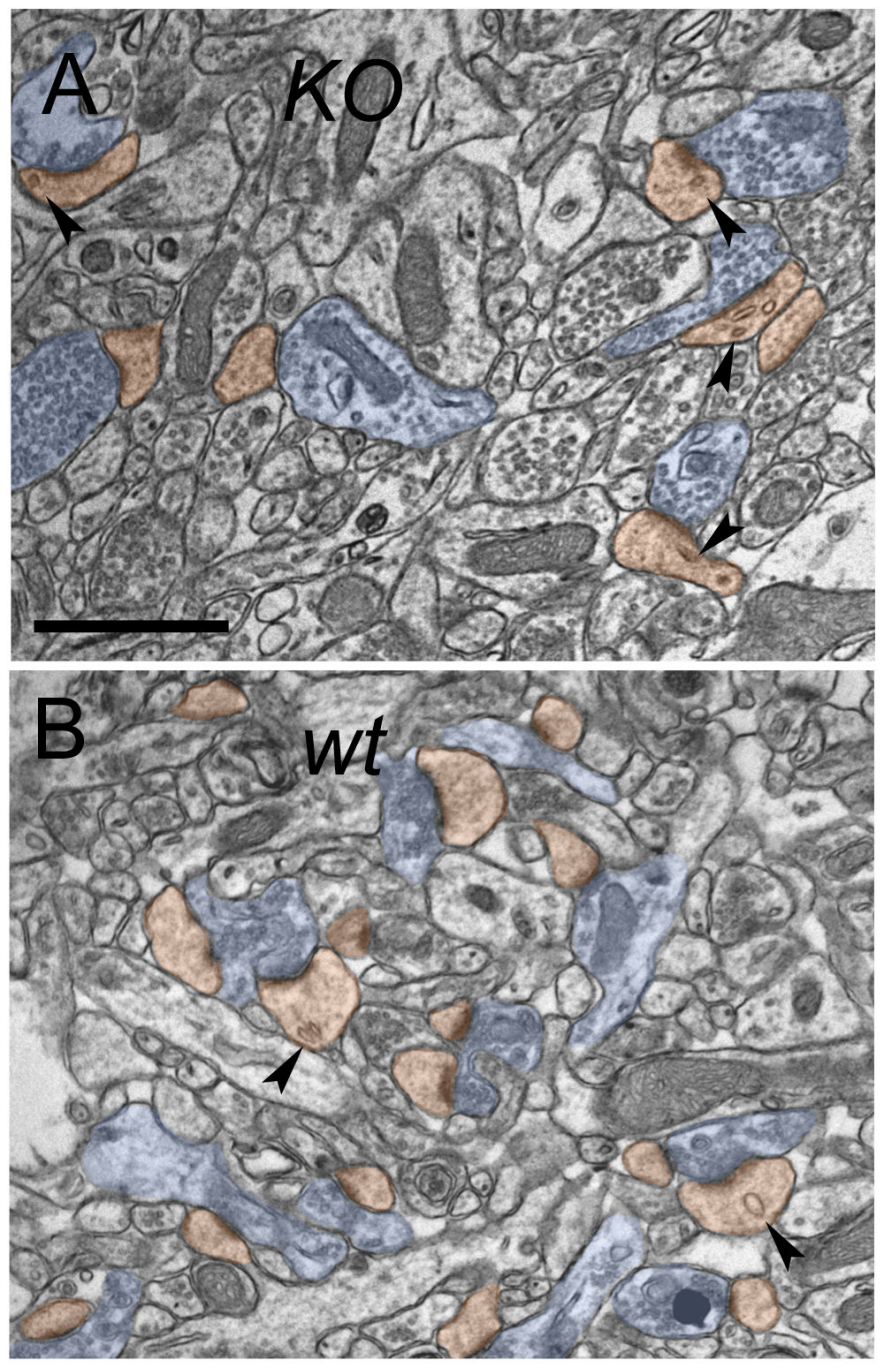
Loss of WAVE-1 causes abnormalities in the internal structure of spines. Representative electron micrographs of CA1 synaptic neuropil; coloring as in Figure 1. Spines from the *KO* material (A) were far more likely to contain endosomes (black arrowheads) than spines from the *wt* (*B*). Scale bar: 1 µm.

We also noticed abnormalities in the internal structure of spines. We found no spine apparatus in our *KO* sample (0 of 989 spines); in contrast, we found that ~2% of spines (35 of 1602) in the *wt* animals had a clearly-defined spine apparatus (typically in large mushroom-shaped spines). On the other hand, spines from the *KO* material contained almost three times more endosomes (46.4 per 100 spines) than spines from the *wt* (16.6); P < 0.001 (Figure 4, arrowheads).

### Synaptic relationship

**Figure 5:**
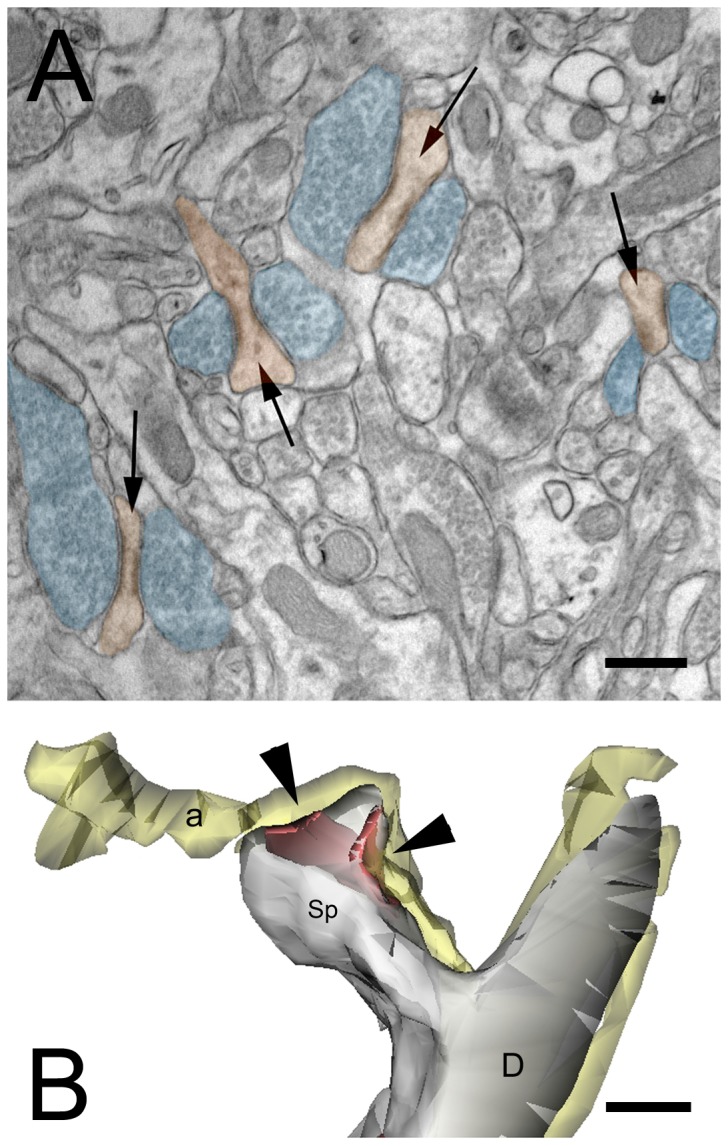
Abnormal spines in stratum radiatum of the WAVE-1 *KO* mouse. **A**. Arrows point to spines with two PSDs. Spines are colored in orange, axon terminals in blue. **B**. 3D reconstruction of an axospinous synaptic contact from mutant hippocampus. The reconstructed CA1 apical dendritic segment (D) has a spine (Sp) oriented to show the synaptic surface. This spine has two distinct postsynaptic densities (red, arrows). The same axon (yellow) establishes separate synaptic contacts with both PSDs. Axon is yellow, spine is grey, PSD is red. Arrows point to synaptic surface between presynaptic active zone and postsynaptic density of the spine (Sp). Scale bars: 200 nm.

The above results suggest that loss of WAVE-1 affects postsynaptic spines more dramatically than axon terminals. Interestingly, we also noted that some of the characteristically flattened spines had two spatially-separated PSDs. These were not typical perforated synapses; the PSDs instead lay on opposite sides of the spine head, apparently contacting two different axon terminals (see arrows, Figure 5A). These features are rarely observed in the normal CA1 stratum radiatum. To see whether these synaptic contacts are established by two independent axon terminals, we performed 3D serial reconstruction of a representative sample of these abnormal synaptic contacts (n= 21), finding that in all cases, a single axon terminal gave rise to both synapses (Figure 5B). These anomalous synaptic contacts might account—at least in part—for the observed numerical asymmetry between the synaptic partners.

In summary, we found that the density of terminals in *KO* animals changed little, but these terminals were bigger, with more but less organized synaptic vesicles. On the postsynaptic side, the density of spines in *KO* animals was significantly reduced, and these spines made abnormal synaptic contacts; furthermore, the spine head was flattened, with an abnormal content of internal membrane-bound structures.

## Discussion

Changes in synaptic efficacy are typically associated with morphological changes, in part because the biochemical cascades implicated in synaptic plasticity share common signaling pathways with the machinery controlling actin dynamics and reorganization [39]. Molecules that serve as ‘hubs’ for these shared pathways are thus essential both for normal neuronal morphology and for activity-dependent synaptic plasticity. Accumulating evidence suggests that WAVE-1 is one such hub: WAVE-1 is required for lamellipodial extension in neuronal growth cones [40], and disruption of the WAVE gene causes deficits at the (glutamatergic) neuromuscular junction in *Drosophila* [41]. Moreover, decreased expression of WAVE-1 (resulting from RNAi) reduces the number of mature dendritic spines in cultured primary hippocampal neurons [29]. Likewise, disruption of upstream signaling to WAVE-1 also causes spine reduction, altered synaptic plasticity, and deficits in memory retention [21,27], as does downstream disruption of the WAVE-1 ligand Abi-2 [42,43] or the Arp2/3 complex [44]. Thus, the WAVE-1 signaling hub appears to play a key role in mediating the morphological changes associated with synaptic plasticity.

The present ultrastructural data from *KO* mice provides clues as to how WAVE-1 may regulate synaptic function in CA1 hippocampus. Presynaptically, we found that loss of WAVE-1 affects the number and distribution of synaptic vesicles in Schaffer-collateral axon terminals in CA1 hippocampus. The biochemical pathway underlying this effect is unclear. Electrophysiological evidence from WAVE-1 *KO* mice revealed that paired-pulse facilitation is normal in the hippocampus, suggesting normal presynaptic release probability [21]. However, phosphorylation of WAVE-1 by cyclin-dependent kinase 5 (Cdk5) inhibits its ability to regulate Arp2/3-dependent actin polymerization, and the functionally recycling vesicle fraction in hippocampal synapses is regulated by Cdk5 activity [45]. Accordingly, we speculate that WAVE-1 in axon terminals may regulate synaptic vesicle distribution via Cdk5.

The abnormal flattening of spine heads in *KO* mice presumably reflects dysregulation of the actin spinoskeleton [10,46,47]. We also found that postsynaptic spines in the *KO* have longer PSDs. Since the length of the PSD correlates with the number of glutamate receptors at the synapse, and the magnitude of EPSCs [48,49,50,51,52], the longer PSDs we detected in the mutants are likely to contain more glutamate receptors, consistent with the enhanced LTP and impaired LTD previously reported for WAVE-1 *KO*s [21]. Current evidence suggests that AMPA receptors are added to the synapse from recycling endosomes in the spine [53,54]. Interestingly we found a marked increase of endosomes within *KO* spines. Whether these endosomes are trapped within the spinoplasm due to defective actin polymerization, or are more numerous because more receptor is being transported to the spine surface (as suggested by the enhanced LTP) will require further investigation.

In conclusion, the combined pre- and postsynaptic changes in synaptic architecture reported here provide a structural substrate for the cognitive deficits previously reported, and support a role for WAVE-1 as an important modulator of synaptic plasticity.

## Supporting Information

Table S1 **Synapse-related parameters for each of the *KO* and *wt* animals studied.** No clear relationship between any of these parameters and sex of mouse was apparent.
(PDF)
