# Supplementary material for: Ultrastructural Abnormalities in CA1 Hippocampus Caused by Deletion of the Actin Regulator WAVE-1
Source: PLoS One. 2013 Sep 25;8(9):e75248. doi: 10.1371/journal.pone.0075248 (PMC3783472; doi:10.1371/journal.pone.0075248)
Supplement: Table S1 — Synapse-related parameters for each of the KO and wt animals studied. No clear relationship between any of these parameters and sex of mouse was apparent. (PDF) [file pone.0075248.s001.pdf]

	Genotype	Animal #	sex	Terminal area (µm^2)	Terminal circularity	N
	KO	5229	F	0.214 ± 0.08	0.68 ± .01	294
	KO	5233	M	0.205 ± 0.08	0.68 ± .01	260
	KO	5266	F	0.195 ± 0.08	0.65 ± .01	196
**Presynaptic terminals**						
	wt	5235	F	0.170 ±0.08	0.66 ± .01	259
	wt	5236	M	0.180 ± 0.06	0.73 ± .01	337
	wt	5264	M	0.169 ± 0.10	0. 64 ± .01	176

	Genotype	Animal #	Sex	Spine area (µm^2)	Spine Circularity	PSD length (nm)	N
	KO	5229	F	0.0716 ± 0.004	0.66 ± .01	250 ± 11	127
	KO	5233	M	0.0817 ± 0.004	0.63 ± .01	270 ± 11	131
	KO	5266	F	0.0764 ± 0.004	0.65 ± .01	258 ± 10	126
**Postsynaptic spines**							
	wt	5235	F	0.0926 ±0.006	0.73 ± .01	224 ± 9	132
	wt	5236	M	0.0769 ± 0.004	0.70 ± .01	219 ± 8	132
	wt	5264	M	0.0686 ± 0.004	0.78 ± .01	204 ± 6	131
